# Weimar Cholera Conference

**Published:** 1867-12

**Authors:** 


### WEIMAR CHOLERA CONFERENCE.

The following from a communication from Dr. Elisha Harris to the President of the New York Board of Health, has been long awaiting a place in our columns:

Having been favored with an abstract of the discussions and concluding recommendations of the Cholera Conference that recently met at the city of Weimar, and having learned from Prof. Pettenkofer that the full stenographic report of the Conference will be published at Leipzic during the summer, I now lay before the Board of Health a synopsis of the discussions and their conclusions as given in this abstract.

You will recollect the polite invitation that was extended to New York to be represented at that important meeting. It turned out to be precisely such a Conference as the interests of public hygiene required, for the most practical and comprehensive questions were discussed by the leading sanitary scholars of Europe, nearly 60 delegates being present. The following conclusions were adopted, and I beg leave to present them here before giving the synopsis of the debates of the Conference.

CONCLUSIONS AND RECOMMENDATIONS.

I. The Conference expresses as its deliberate conviction that the efforts to arrest and prevent cholera by disinfectants, should be continued in the most energetic manner.

II. Disinfection will be entirely successful only where excremental matters are carefully gathered and kept from being cast about; when attention is given to the cleanliness and the means of health; and when the disinfection is performed by sanitary authorities in a compulsory manner.

III. In places where the entire locality or district cannot at once be disinfected, it is advisable to disinfect throughout the places visited by the previous epidemics of cholera.

IV. The general disinfection should be performed at the proper time, that is, before the epidemic is actually prevalent in town or place. Every house or spot that becomes infected or is suspected to be so, must be kept constantly under the influence of disinfection.

V. In regard to the best substances as disinfectants, though the testing of various articles is not completed, there have been found, to the present time, no more effectual substances than sulphate of iron (copperas) and the carbolic acid; and, as experience proves, we have no other disinfectants that can be employed with greater facility. A combination of both these disinfectants is therefore recommended.

VI. The disinfection of clothing that has become infected by cholera excrement is especially an important matter. For that purpose the Conference recommends that all such clothing be disinfected by boiling in water, or by chemical treatment in a proper solution of "zinc vitrol" (sulphate or chloride of zinc), and the Conference also recommends that special arrangements be made by which disinfection can be employed in all places, and at any hour, among or for the poor.

VII. For the disinfection of sewers and drains, the Conference advises the trial of Mr. Sauvren's method. [The means used by Mr. Sauvren are not yet fully published, but they are believed to be similar to McDougall's—namely, combination of a carbolic or coal-tar preparations, in a cheap form.]

VIII. If cholera infects any house or spot, it is recommended that, if practicable, the houses so situated in an infected place, or being infected, should be vacated, and their inhabitants should be removed from the infected spot.

IX. It is especially recommended that the ground-water (that is the water in the ground) about dwelling-houses, and all the grounds about habitations of every kind, should be preserved undefiled by any excremental matter of cholera; also, that all drinking water be undefiled and pure, and that where no pure water can be had that the water which must be used should be disinfected by boiling.

Such were the final conclusions of the Conference in reference to the first duties of sanitary authorities, and the people of any town that is threatened by cholera. The discussions were based upon the experience and studies of the distinguished gentlemen who had thus agreed to meet and compare their views, and the results of their observations. The attendants at the conference were from various cities of Germany, Holland, Prussia, Austria, Hungary, and Russia. The history of cholera outbreaks among the troops in the war last year proved marvellously interesting, and conclusive on many points. Next in order of interest and importance was the history of infection by means of water contaminated by cholera excrement. Closely allied to the latter subject was the examination of evidence concerning the discoveries that have been made in regard to the particular means by which the cholera infection is transported and propagated. Lastly and most practically useful was the examination of evidences concerning the proper and best methods of disinfection, and the relations of such means to the control and promotion of cholera epidemics. The chief medical officer to the Privy Council of Great Britain presented the history of the outbreaks of cholera in London in connection with the water of the East London Water Company, which, as Dr. Radcliffe has shown, was contaminated by cholera excrement. In the district where that water was used the epidemic burst forth as by explosion; while, subsequently, in other places it spread by the more usual methods and in the more usual manner. Then again, there were other instances where the epidemic spared all persons in certain asylums and hospitals who used privies that were entirely uncontaminated by cholera excrement, while the epidemic decimated the classes of inmates that used the latter. The Conference conceded that wells and reservoirs of drinking water were frequently contaminated by the cholera poison by soakage into them of the infectious element from the cholera stools; but Profs. Pettenkofer, Wanderlich, Simon, and others, agreed that drinking water was not the most universally common means of communicating cholera to man. The influence of ground-moisture, or more precisely of the ordinary ground-water, while such water or moisture is receding by drying of the ground after a wet period, was proved by such who daily used the same well water, but who used different privies and frequented different and well separated yards, as we saw the same fact illustrated in two adjacent pavilions on Blackwell's Island, last summer. The influence of different kinds of ground in receiving and propagating the epidemic virus of cholera was examined, and Dr. Pfeiffer, of Vienna, showed the curious course which the epidemic pursued in passing through the great forest country of Thuringen last year; while the delegates from Dresden and some other places showed what conditions of the earth had permitted and favored the spread of cholera on their soil, that covered certain granite rock districts. The outbreaks on Blackwell's Island and the rocky summit of Hudson City, fully bear out the conclusions of the Conference on the subject of cholera epidemics on rocky surfaces, and do not disprove the agency of the surface soil in propagating the virus when planted in such places. Examining the great mass of facts presented by members of the Conference in regard to influence of the ground and its retentiveness of undrained water or of being porous, and, at times saturated and again undergoing a course of drying by evaporation, the more important conclusions seem to be as follows:

1. That porous soils, and any kind of earth that retains and favors the ordinary kinds of fermenting filth, will readily retain and repropagate the virus of cholera when once the germinal virus has been introduced or planted by persons coming from infected places. That the mere altitude of a place is not the question that determines its susceptibility to cnolera; that the moisture (ground-water) and the fluctuations of that moisture of a soil by rising and receding (drying), favor the propagation of cholera; that a sewer or drain may become the chief source of infection to some places where there is no soil, or where the ground and everything except the sewers and drains have been disinfected.

2. That Prof. Pettenkofer's use of the term ground-water should be understood, as he intended, to mean the standard of saturation by moisture in the soil, and that grounds which, upon their surface appear to be high and dry may, nevertheless, be saturated with moisture; that is, have an excess of ground-water (or high ground-water), and that the Sanitary drainage and drying which are necessary to protect a soil against repropagating the planted virus or germs of cholera must be deep and thorough. The history and topography of the cholera fields of Halle, Berlin, Zwickeon, Thuringen, Helsingfors, and St. Petersburg supplied admirable proofs of this great doctrine in sanitary drainage.

3. Good proofs were adduced that there are some kinds of soil that seem to be natural disinfectants of cholera virus, and upon which an epidemic cannot spread except in filthy houses, sewers, etc. We have not time to make the abstract of the facts that will illustrate the true theory of this kind of exemption. We can say, however, that it is plainly important that regard should be given to the kinds of earth and materials used for filling up sunken lots, and that even the location of dwelling-places may sometimes be wisely a matter of choice as regards the nature of the soil.

The facts concerning specific disinfection to destroy both the cholera virus and all susceptibility to material for its repropagation in a house or a district were well discussed in the Conference. The negative facts were specially important, for they showed that in a few places, as in the great prison at Halle, the epidemic swept forward regardless of the previous and continued disinfection of the grounds and nuisances with sulphate of iron. But in those instances it was proved that the sewers and drains were not disinfected, and that not only were the infected spots particularly exposed to and connected with such drains and sewers, but that the copperas solution had been relied upon without admixture with carbolic acid, and the powerfnl antiseptic agents which coal-tar contains. In Berlin there was great success in the use of permanganate of soda, with sulphuric acid added—that is, the success was achieved by the most rapid and powerful oxidization, in the same manner as we last summer disinfected the defiled clothing and bedding of the cholera sick by means of permanganate of potassa. The expensiveness of the method is the chief objection to it. Yet, for domestic and limited applications, it is a perfect method for clothing and upholstery. The fact that with entire unanimity the Conference recommended that the main reliance for disinfection should be placed in the simpler and powerful agents—sulphate of iron and carbolic acid, which the Metropolitan Board unhesitatingly adopted at the beginning of the epidemic last year—will be amply warrant for out continuing to employ those cheap and effectual substances.

The vital importance of perfect sanitary care of all persons sick or infected with cholera, was illustrated in the history of the epidemic in every city. Disinfection alone, especially the irregular and unsystematic or unenforced applications of disinfection, did not always control the prevalence of cholera; indeed, such exclusive and unmethodical sanitary work often resulted in fatal disappointments. In some cities, as in Erfurth, even the carbolic acid was so freely used in some parts of the town (in privies), that the wells in the vicinity of privies flooded with that disinfectant, yielded water that tasted strongly of it; yet parts of Erfurth were neglected, and cholera was fearfully epidemic there. But it was conceded that in cities in which there was perfect, and systematic and well-regulated sanitary disinfection, combined with perfect care of the sick and of all suspected persons, as was the case in the city of Bristol and some other favorite cholera haunts, the epidemic was controlled, and, by like faithfulness and skill, that it could and should be generally controlled in all civilized cities.

Professor Hirsch presented the arguments and studies that favor the discovery of the precise nature of the poison that produces cholera, and the Conference commended and urged on the inquiries that have already, in the hands of Professors Klob and Thome, last year, resulted in discovering a minute microscopical growth that seems, thus far, to be exclusively produced in cholera excrements, and which obey all the tests for the destruction as well as the propagations of the cholera. The spores of that little growth multiply with marvellous rapidity, and they are not destroyed by ordinary doses of chlorine or chloride of lime, but are killed by sulphate of iron and carbolic acid.

The Conference recommend that scientific naturalists, like the men who are now at work on these questions, should continue their researches. It was also recommended that observers of cholera should carefully study the conditions under which the epidemic is transported from place to place, and also study the relations of grounds, moisture (ground-water), and other local conditions that determine the bounderies of epidemic fields.

It will be observed by these notes, of a discussion that appears to have been conducted with the single object to find out what is known, that there was a clear knowledge of the practical wants of sanitary officers and governments. The nine propositions which the members of Conference have submitted as their unanimous conclusions and recommendations, I have placed at the beginning of this abstract, as being precisely the kind of information which a Board of Health most wishes to receive, and upon which it can base judicious practices. Fortunately for the good name of your Board as for the safety of the city last year, our practice was, from the first, based upon these doctrines, and the great minds that led in the Weimar Conference were the men that had most aided us in former years to deal with epidemic and infectious diseases. I am happy to learn that the Leipzig report is to be fully illustrated by maps and charts to show precisely what course cholera has pursued in European cities. We may hope to receive copies next month. I regret that the Abstract forwarded by Professor Pettenkofer cannot be entirely translated and placed in your hands to-day. These pages contain the gist of the whole, but the debates touched upon a great many other points. We are a little surprised that some conclusion and recommendation on quarantine was not reached. But, since the fact has been demonstrated that persons who travel away from an infected district may themselves, while yet journeying and not sick, spread cholera, by means of excremental evacuations, it is not surprising that little reliance should be placed upon quarantine regulations as a means of preventing cholera from spreading in Europe.—*Phila. Medical & Surgical Reporter.*

